# Supplementary material for: Estimating time of HIV-1 infection from next-generation sequence diversity
Source: PLoS Comput Biol. 2017 Oct 2;13(10):e1005775. doi: 10.1371/journal.pcbi.1005775 (PMC5638550; doi:10.1371/journal.pcbi.1005775)

Fig S3. Coefficient of determination for different diversity measures, including all sites. (Genetic region: *pol*.)

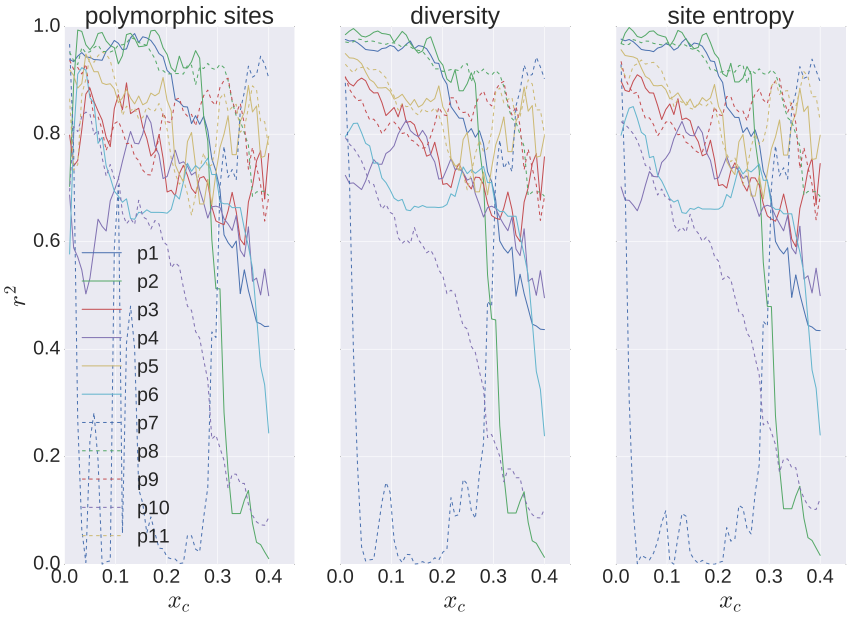

Supplement: S3 Fig — (Genetic region: pol.) (PDF) [file pcbi.1005775.s003.pdf]
