# Supplementary material for: Estimating time of HIV-1 infection from next-generation sequence diversity
Source: PLoS Comput Biol. 2017 Oct 2;13(10):e1005775. doi: 10.1371/journal.pcbi.1005775 (PMC5638550; doi:10.1371/journal.pcbi.1005775)

**Fig S5. Mean absolute error as a function of the low-frequency cutoff ($x_c$).** Different diversity measures perform very similarly when the cutoff $x_c$ is large. Average pairwise distance and entropy outperform fraction of polymorphic sites for low $x_c$. This graph is based on diversity in *gag* (left) and *env* (Right). Solid lines correspond to using all sites, dashed - only 3rd codon positions.

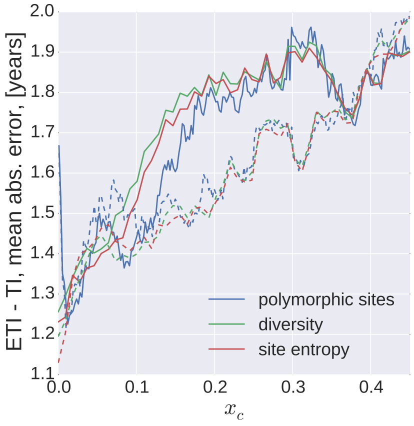


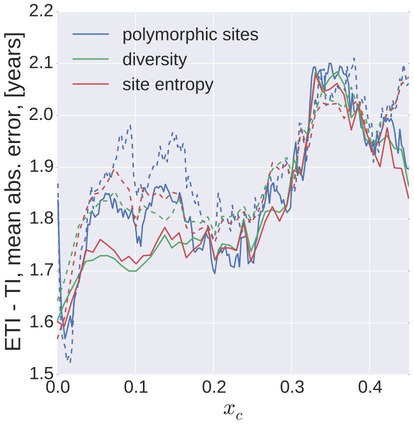

Supplement: S5 Fig — Different diversity measures perform very similarly when the cutoff xc is large. Average pairwise distance and entropy outperform fraction of polymorphic sites for low xc. This graph is based on diversity in gag (left) and env (Right). Solid lines correspond to using all sites, dashed—only 3rd codon positions. (PDF) [file pcbi.1005775.s005.pdf]
