# Supplementary material for: Estimating time of HIV-1 infection from next-generation sequence diversity
Source: PLoS Comput Biol. 2017 Oct 2;13(10):e1005775. doi: 10.1371/journal.pcbi.1005775 (PMC5638550; doi:10.1371/journal.pcbi.1005775)

Fig S6. Distribution of slopes (s) and intercepts (t_0) (bootstrapped over the patients of the training dataset). (Genetic region: 3rd codon positions in *pol*, diversity measure: average pairwise distance, $x_c = 0.003$.)

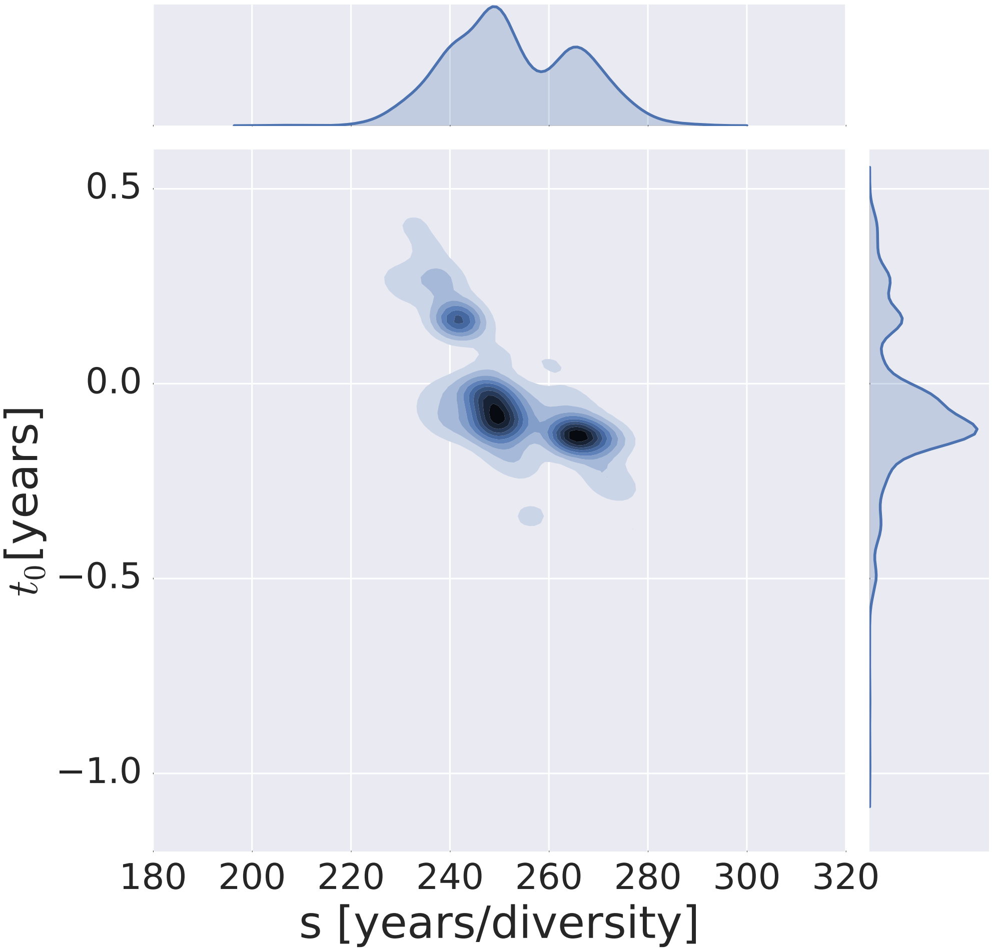

Supplement: S6 Fig — (Genetic region: 3rd codon positions in pol, diversity measure: average pairwise distance, xc = 0.003.) (PDF) [file pcbi.1005775.s006.pdf]
