# Supplementary material for: Estimating time of HIV-1 infection from next-generation sequence diversity
Source: PLoS Comput Biol. 2017 Oct 2;13(10):e1005775. doi: 10.1371/journal.pcbi.1005775 (PMC5638550; doi:10.1371/journal.pcbi.1005775)

Fig S10. (Left) Distribution of the estimation error. (Right) Estimated time of infection (ETI) versus actual time of infection (TI). Displayed for the training and the validation data sets. (Genetic region: 3rd codon positions in *gag*, diversity measure: average pairwise distance, $x_c = 0.003$.)

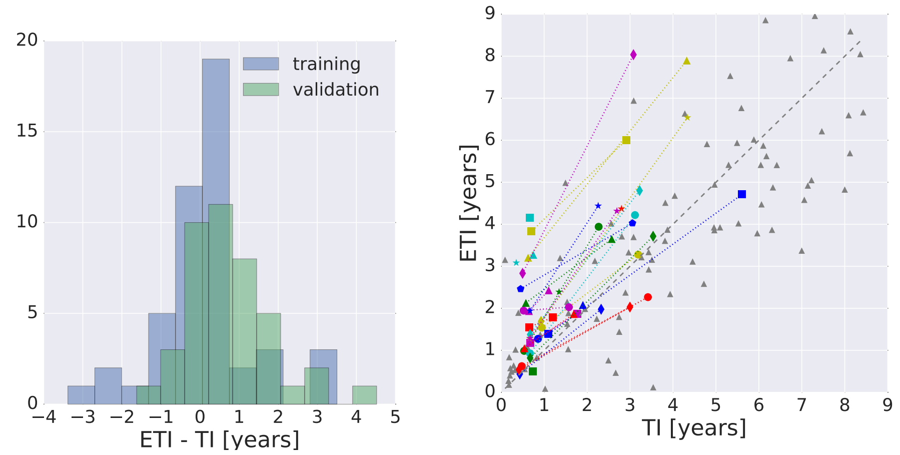

Supplement: S10 Fig — (Genetic region: 3rd codon positions in gag, diversity measure: average pairwise distance, xc = 0.003. The encircled outliers are discussed in the text.) (PDF) [file pcbi.1005775.s010.pdf]
