# Supplementary material for: Estimating time of HIV-1 infection from next-generation sequence diversity
Source: PLoS Comput Biol. 2017 Oct 2;13(10):e1005775. doi: 10.1371/journal.pcbi.1005775 (PMC5638550; doi:10.1371/journal.pcbi.1005775)

Fig S11. Estimated time of infection (ETI) versus actual time of infection (TI). Displayed for the training and the validation data sets. Legend shows the subtype (top) and transmission route (bottom) for the validation dataset patients. (Genetic region: 3rd codon positions in *pol*, diversity measure: average pairwise distance, $x_c = 0.003$.)

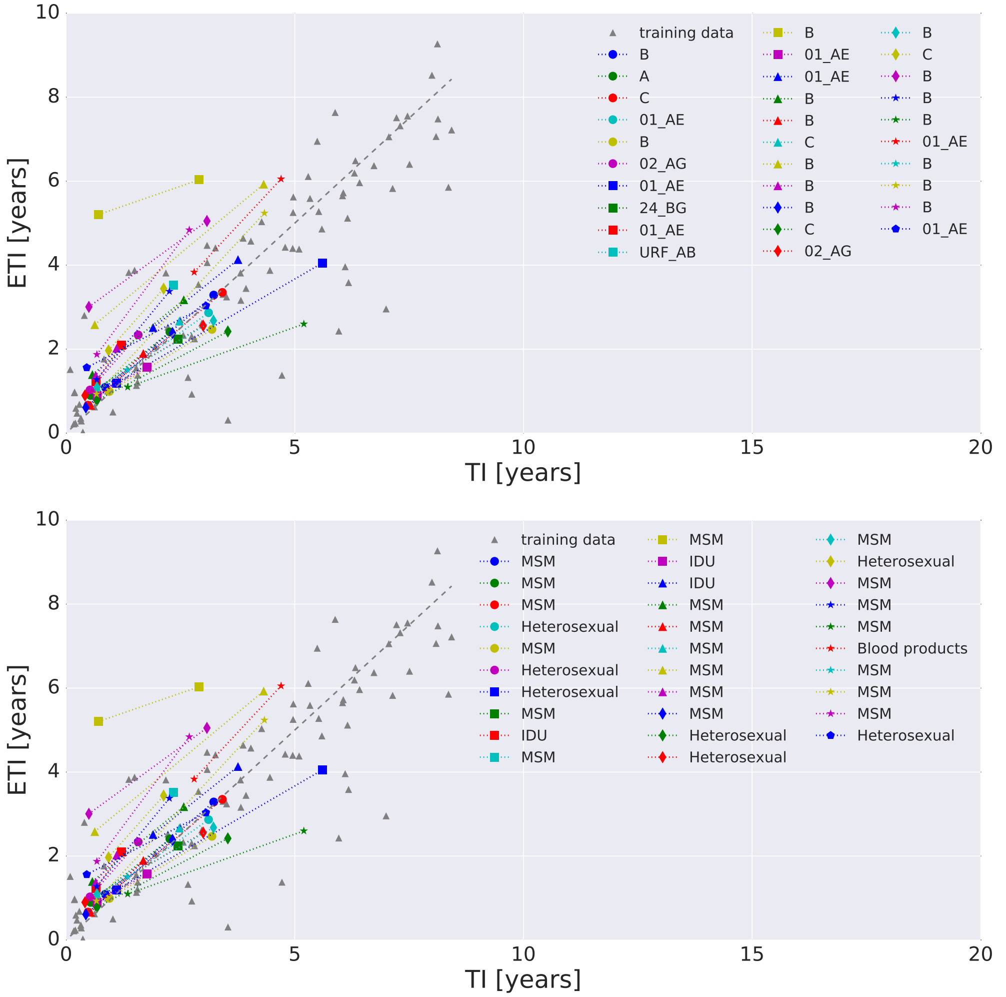

Supplement: S11 Fig — (Genetic region: 3rd codon positions in pol, diversity measure: average pairwise distance, xc = 0.003.) (PDF) [file pcbi.1005775.s011.pdf]
