# Supplementary material for: Estimating time of HIV-1 infection from next-generation sequence diversity
Source: PLoS Comput Biol. 2017 Oct 2;13(10):e1005775. doi: 10.1371/journal.pcbi.1005775 (PMC5638550; doi:10.1371/journal.pcbi.1005775)
Supplement: S1 Appendix — (PDF) [file pcbi.1005775.s013.pdf]

S1 Appendix Linear fitting procedures Given a set of K time points (t_k) and corresponding values of mean/average diversity (D_k) we infer slope and intercept pair (s, t_0) by performing linear regression. Here we used least absolute deviation (LAD) method, which means minimizing the mean absolute deviation (MAE)

$$S_{LAD} = \frac{1}{K}\sum_{k=1}^{K} |sD_k + t_0 - t_k| \tag{1}$$

in respect to parameters s and t_0;.

LAD has an advantage over the traditional optimal least squares approach (OLS) in that it is less sensitive to the possible outliers. Minimization can be done either using one of the efficient linear programming techniques, or by considering all pairs s, t_0 corresponding to straight lines passing through all possible pairs of data points, and choosing the pair corresponding to the least error. (It is known that the regression line corresponding to the minimum of S_{LAD} passes through at least two of the data points.) We used the latter approach, as it is quite efficient for rather small number of data points that we had to deal with, but also could be modified to perform in parallel calculations for several genetic regions, which was useful for the sliding window analysis of the error along the genome.
