# Supplementary material for: Estimating time of HIV-1 infection from next-generation sequence diversity
Source: PLoS Comput Biol. 2017 Oct 2;13(10):e1005775. doi: 10.1371/journal.pcbi.1005775 (PMC5638550; doi:10.1371/journal.pcbi.1005775)
Supplement: S2 Appendix — (PDF) [file pcbi.1005775.s014.pdf]

S2 Appendix Moving average The moving average for the error dependence on the time of infection was calculated using the mean absolute error for a set of n points:

$$S_q = \frac{1}{n} \sum_{k=q+1}^{q+n} |sD_k + t_0 - t_k| \tag{2}$$

while the corresponding times of infection where evaluated as

$$\tau_q = \frac{1}{n} \sum_{k=q+1}^{q+n} t_k. \tag{3}$$

The advantage of this technique over other time averaging approaches is that one avoids significant variation of the averaging error, since the number of data points in the average is fixed to n.
