# Supplementary material for: Estimating time of HIV-1 infection from next-generation sequence diversity
Source: PLoS Comput Biol. 2017 Oct 2;13(10):e1005775. doi: 10.1371/journal.pcbi.1005775 (PMC5638550; doi:10.1371/journal.pcbi.1005775)
Supplement: S1 Table — (Genetic region: 3rd codon positions in pol, diversity measure: average number of polymorphic sites. ain years/diversity; bin years.) (PDF) [file pcbi.1005775.s015.pdf]

S1 Table Recommended slope and intercept values depending on the cutoff.

	slope and intercept			slope only	
cutoff (x_c)	slope (s)^a	intercept (t_0)^b	MAE^b	slope (s)^a	MAE^b
0.05	78.16	0.12	1.02	80.50	1.01
0.10	120.79	0.40	1.01	129.92	1.05
0.15	168.42	0.52	1.05	183.90	1.11
0.20	211.29	0.70	1.18	233.76	1.23
0.25	253.30	1.03	1.32	312.03	1.43
0.30	328.22	1.09	1.45	404.20	1.60
0.35	381.41	1.56	1.59	565.60	1.72
0.40	603.82	1.54	1.68	834.63	1.85
0.45	694.28	2.53	1.87	1157.02	2.62

Genetic region: 3rd codon positions in *pol*, diversity measure: average number of polymorphic sites. ^{a}in years/diversity; ^{b}in years.
