# Supplementary material for: Estimating time of HIV-1 infection from next-generation sequence diversity
Source: PLoS Comput Biol. 2017 Oct 2;13(10):e1005775. doi: 10.1371/journal.pcbi.1005775 (PMC5638550; doi:10.1371/journal.pcbi.1005775)
Supplement: S2 Table — (Genetic region: 3rd codon positions in pol, diversity measure: average site entropy ain years/diversity; bin years.) (PDF) [file pcbi.1005775.s016.pdf]

S2 Table Recommended slope and intercept values depending on the cutoff.

	slope and intercept			slope only	
cutoff (x_c)	slope (s)[a]	intercept (t_0)[b]	MAE[b]	slope (s)[a]	MAE[b]
0.00	196.34	-0.19	0.85	189.31	0.85
0.05	256.99	0.26	0.95	271.60	0.95
0.10	321.13	0.41	1.01	350.22	1.04
0.15	387.34	0.70	1.11	447.40	1.15
0.20	465.61	0.74	1.21	526.40	1.27
0.25	518.50	1.02	1.34	678.49	1.44
0.30	674.63	1.18	1.46	831.50	1.61
0.35	773.04	1.54	1.59	1170.50	1.72
0.40	1211.36	1.54	1.70	1676.82	1.85
0.45	1392.68	2.54	1.87	2310.37	2.61

Genetic region: 3rd codon positions in *pol*, diversity measure: average site entropy [a]in years/diversity; [b]in years.
