# Supplementary material for: Estimating time of HIV-1 infection from next-generation sequence diversity
Source: PLoS Comput Biol. 2017 Oct 2;13(10):e1005775. doi: 10.1371/journal.pcbi.1005775 (PMC5638550; doi:10.1371/journal.pcbi.1005775)
Supplement: S3 Table — (Genetic region: all sites in pol, diversity measure: average pairwise distance. ain years/diversity; bin years.) (PDF) [file pcbi.1005775.s017.pdf]

S3 Table Recommended slope and intercept values depending on the cutoff.

	slope and intercept			slope only	
cutoff (x_c)	slope (s)^a	intercept (t_0)^b	MAE^b	slope (s)^a	MAE^b
0.00	574.51	-0.52	0.85	517.61	0.88
0.05	691.67	-0.02	0.94	673.42	0.92
0.10	795.07	0.11	0.91	830.76	0.92
0.15	972.00	0.21	1.02	1029.23	1.03
0.20	1111.40	0.32	1.10	1217.34	1.10
0.25	1292.90	0.57	1.20	1434.69	1.25
0.30	1547.65	0.79	1.30	1788.47	1.37
0.35	1987.89	0.92	1.43	2531.70	1.46
0.40	2415.57	1.31	1.68	3345.15	1.75
0.45	3131.26	2.23	1.85	5204.78	2.27

Genetic region: all sites in *pol*, diversity measure: average pairwise distance. ^{a}in years/diversity; ^{b}in years.
