# Supplementary material for: Estimating time of HIV-1 infection from next-generation sequence diversity
Source: PLoS Comput Biol. 2017 Oct 2;13(10):e1005775. doi: 10.1371/journal.pcbi.1005775 (PMC5638550; doi:10.1371/journal.pcbi.1005775)
Supplement: S4 Table — (Genetic region: all sites in pol, diversity measure: average number of polymorphic sites. ain years/diversity; bin years.) (PDF) [file pcbi.1005775.s018.pdf]

S4 Table Recommended slope and intercept values depending on the cutoff.

	slope and intercept			slope only	
cutoff (x_c)	slope (s)^a	intercept (t_0)^b	MAE^b	slope (s)^a	MAE^b
0.05	181.68	-0.17	1.08	171.09	1.10
0.10	283.41	0.10	0.90	287.39	0.88
0.15	389.90	0.17	0.96	401.41	0.96
0.20	490.18	0.28	1.07	528.22	1.09
0.25	587.25	0.57	1.21	652.39	1.23
0.30	734.72	0.78	1.30	862.30	1.36
0.35	968.38	0.88	1.44	1229.70	1.45
0.40	1208.07	1.26	1.70	1663.00	1.77
0.45	1595.22	2.23	1.82	2599.17	2.27

Genetic region: all sites in *pol*, diversity measure: average number of polymorphic sites.
^{a}in years/diversity; ^{b}in years.
