# Supplementary material for: Estimating time of HIV-1 infection from next-generation sequence diversity
Source: PLoS Comput Biol. 2017 Oct 2;13(10):e1005775. doi: 10.1371/journal.pcbi.1005775 (PMC5638550; doi:10.1371/journal.pcbi.1005775)
Supplement: S5 Table — (Genetic region: all sites in pol, diversity measure: average site entropy. ain years/diversity; bin years.) (PDF) [file pcbi.1005775.s019.pdf]

S5 Table Recommended slope and intercept values depending on the cutoff.

	slope and intercept			slope only	
cutoff (x_c)	slope (s)[a]	intercept (t_0)[b]	MAE[b]	slope (s)[a]	MAE[b]
0.00	445.61	-0.75	0.91	390.96	0.93
0.05	595.29	-0.12	0.97	582.31	0.95
0.10	745.57	0.07	0.89	755.98	0.88
0.15	909.35	0.19	0.99	944.97	1.00
0.20	1089.94	0.29	1.09	1160.02	1.10
0.25	1236.10	0.57	1.20	1370.86	1.24
0.30	1511.44	0.79	1.29	1772.83	1.35
0.35	1941.61	0.89	1.42	2456.16	1.44
0.40	2405.31	1.35	1.66	3229.49	1.74
0.45	3062.32	2.28	1.84	5200.83	2.26

Genetic region: all sites in *pol*, diversity measure: average site entropy. [a]in years/diversity; [b]in years.
