# Supplementary material for: Estimating time of HIV-1 infection from next-generation sequence diversity
Source: PLoS Comput Biol. 2017 Oct 2;13(10):e1005775. doi: 10.1371/journal.pcbi.1005775 (PMC5638550; doi:10.1371/journal.pcbi.1005775)

Fig S12. Estimated time of infection (ETI) versus actual time of infection (TI). Displayed for the training and the validation data sets. Legend shows the number of templates (top) and the dilutions (bottom) for the validation dataset. (Genetic region: 3rd codon positions in *pol*, diversity measure: average pairwise distance, $x_c = 0.003$.)

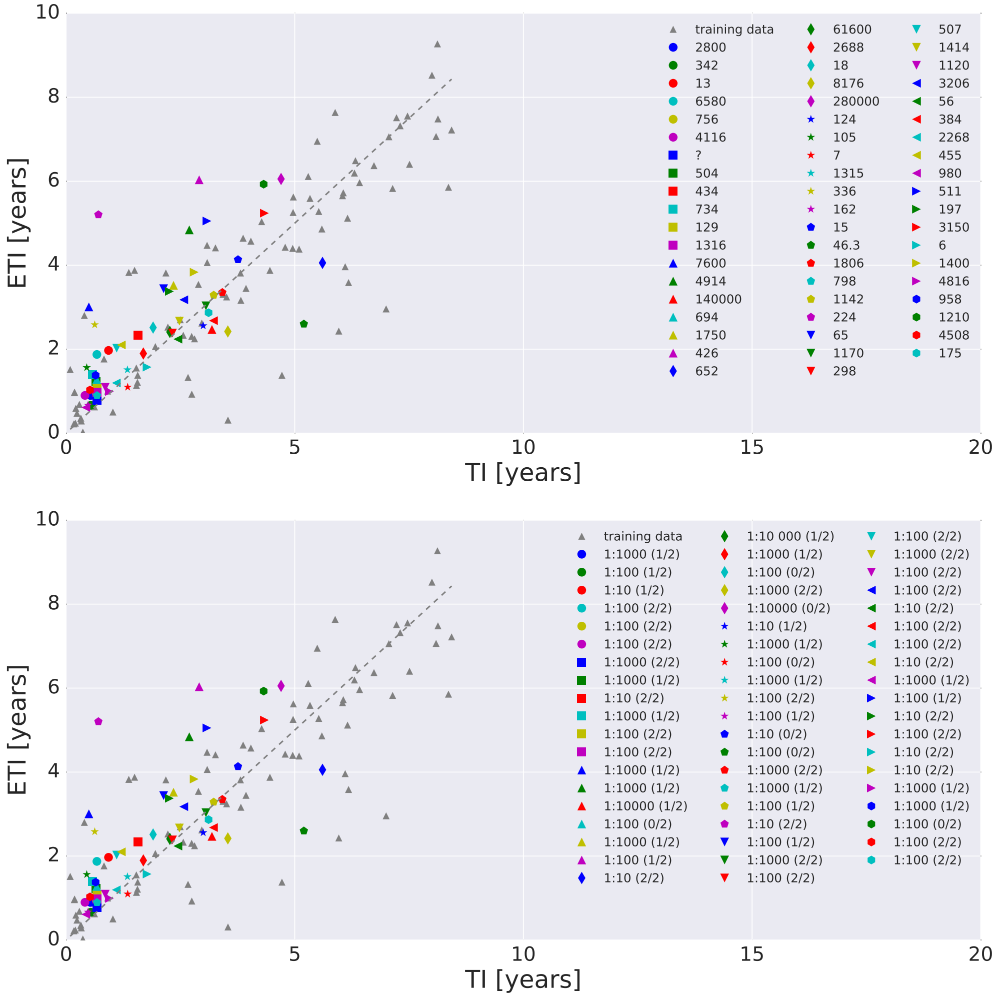

Supplement: S12 Fig — (Genetic region: 3rd codon positions in pol, diversity measure: average pairwise distance, xc = 0.003.) (PDF) [file pcbi.1005775.s012.pdf]
