# Supplementary material for: Estimating time of HIV-1 infection from next-generation sequence diversity
Source: PLoS Comput Biol. 2017 Oct 2;13(10):e1005775. doi: 10.1371/journal.pcbi.1005775 (PMC5638550; doi:10.1371/journal.pcbi.1005775)
Supplement: S1 Text — (PDF) [file pcbi.1005775.s022.pdf]

Comments to supplement table on estimated "true" time of infection

Patient 1
TI was based on a combination Fiebig staging and BED test results, because he patient was in Fiebig stage VI (i.e. >100 days since TI), whereas BED testing on a sample obtained 22 days later indicated a shorter time (85 days) since TI. Based on these data TI was placed 100 days before date for HIV serology for Fiebig staging. The first sequenced sample was obtained 122 days after TI.
The first VL was <500 and was obtained 122 days after TI. This VL may be false low because the first-generation Roche HIV monitor test, which was used until fall of 1997, is known to sometimes under-quantify non-B-subtypes including CRF01_AE (Alaeus, AIDS Res Hum Retroviruses 1999;15:889).

Patient 2
TI was based on Fiebig stage V. BED also indicates early infection (61 days).The first sequenced sample was obtained 74 days after TI.

Patient 3
Fiebig stage VI indicated >100 days since TI. Therefore, TI was based on the BED test result that indicated that the first sample used for sequencing (and BED testing) was obtained 146 days after TI.

Patient 4
TI was based on Fiebig V. Based on this TI the first sequenced sample was obtained 93 days after TI. BED agrees quite well with this TI.

Patient 5
TI was based on a combination Fiebig staging and BED test results, because he patient was in Fiebig stage VI (i.e. >100 days since TI), whereas BED testing on the same sample indicated a shorter time (60 days) since TI. Based on these data TI was placed 100 days before date for HIV serology for Fiebig staging. The first sequenced sample was obtained 134 days after TI and had a VL of <50.

Patient 6
TI was based on a laboratory-confirmed primary HIV infection (PHI). Fiebig staging and BED agrees relatively well with this TI. Note that Fiebing staging was based on RIBA testing instead of Western blot (WB) because no sample remained for retrospective WB testing. The first sample for sequencing was obtained 62 days after TI and had a VL of 6800.

Patient 7
TI was based on the midpoint between the last negative and the first positive HIV-1 test, which were obtained 190 days apart. The patient was in Fiebig stage VI at diagnosis and no BED test result was available.

Patient 8
TI was based on Fiebig V. Based on this TI the first sequenced sample was obtained 87 days after TI. BED agrees quite well with this TI.

Patient 9
TI was based on a combination of Fiebig staging and BED test results. Fiebig stage VI indicated >100 days since TI, whereas BED testing on a sample obtained 6 days later indicated a shorter time (71 days) since TI. Based on these data TI

was placed 100 days before the date of sampling for Fiebig staging, which meant that the first sample used for sequencing was obtained 106 days after TI.

Patient 10

TI was based on laboratory confirmed primary HIV infection. TI based on Fiebig staging and BED agrees quite well with this TI. The first sample for sequencing was obtained 33 days after TI.

Patient 11

TI was based on a combination of Fiebig staging and BED test results. Fiebig stage VI indicated >100 days since TI, whereas BED testing on a sample obtained 16 days later indicated 208 days since TI. Based on these data TI was placed 208 days before the date of sampling for BED testing. This was also the first sample used for sequencing.
